# Neuroprotective effect of Azadirachta indica standardized extract in partial sciatic nerve injury in rats: Evidence from anti-inflammatory, antioxidant and anti-apoptotic studies

**DOI:** 10.17179/excli2017-161

**Published:** 2017-04-18

**Authors:** Amit D. Kandhare, Anwesha A. Mukherjee, Subhash L. Bodhankar

**Affiliations:** 1 Department of Pharmacology, Poona College of Pharmacy, Bharati Vidyapeeth Deemed University, Erandwane, Paud Road, Pune-411 038, India

**Keywords:** Azadirachta indica, partial sciatic nerve ligation, neuropathic pain, NF-kappaB, BAX, caspase-3, apoptosis, flow cytometric analysis, motor nerve conduction velocity

## Abstract

Chronic neuropathic pain is a common and widely recognized pain syndrome for patients and difficult to manage for physicians. *Azadirachta indica *(AI) possesses analgesic, anti-inflammatory, and antioxidant properties. To evaluate the neuroprotective effect of AI standardized extract in an animal model of peripheral neuropathy induced by partial sciatic nerve ligation (PSNL). PSNL was induced in male Wistar rats (180-200 g) with tight ligation of the nerve. Rats received treatment with either vehicle i.e. distilled water (PSNL control), Pyridoxine (100 mg/kg, p.o.) or AI (100, 200 and 400 mg/kg, p.o.) for 28 days. Various behavioral parameters, biochemical, molecular and histological parameters were evaluated. PSNL resulted in a significant decrease (*p < *0.05) in allodynia, hyperalgesia, motor coordination and motor nerve conduction velocity (MNCV) whereas chronic treatment with AI (200 and 400 mg/kg) significantly attenuated (*p < *0.05) these behavioral changes. Enhanced activity of oxidative-nitrosative stress, inflammatory mediators (TNF-α, IL-1β, and NF-κB) as well as mRNA expression of Bax, Caspase-3, and iNOs were significantly attenuated (*p < *0.05) by AI treatment. It also significantly increased (*p < *0.05) peripheral blood oxygen content and Bcl-2 mRNA expression. The flow cytometric analysis revealed that AI (200 and 400 mg/kg) treatment significantly attenuated neural apoptosis and reactive oxygen species levels. PSNL induced histological aberrations were also decreased by AI treatment. *Azadirachta indica* exerts its neuroprotection against PSNL induced neuropathic pain via inhibition of oxidative-nitrosative stress, the release of pro-inflammatory cytokines and apoptosis to improve MNCV (graphical abstract, Figure 1(Fig. 1)).

## Abbreviations

*Azadirachta indica* (AI); Bcl-2-associated X protein (BAX); B-cell lymphoma 2 (Bcl-2); Central Nervous System (CNS); Cyclooxygenase-2 (COX-2); Reduced glutathione (GSH); Interleukin-1 Beta (IL-1β); Inducible nitric oxide synthase (iNOs); Malondialdehyde (MDA); Motor Nerve Conduction Velocity (MNCV); Nuclear factor-kappaB (NF-κB); Polymerase Chain Reaction (PCR); Partial Sciatic Nerve Ligation (PSNL); Reactive Oxygen Species (ROS); Superoxide Dismutase (SOD); Tumor Necrosis Factor-alpha (TNF-α)

## Introduction

Neuropathic pain refers to pain as a result of damage (due to injury or disease) to the nervous system including nerves, spinal cord, and certain central nervous system (CNS) regions (Zimmermann, 2001[88]) with heterogeneous etiology and the complex pathophysiology (Woolf and Mannion, 1999[86]). Patients with diabetes, cancer, AIDS, leprosy, cervical disc protrusion and foraminotomy mainly suffer from peripheral neuropathic pain (Garg and Dehran, 2010[25]; Shaladi et al., 2009[74]). According to World Health Organization, around 22 % of the world's primary care patients suffer from chronic neuropathic pain and there is an urgent need to address this by all physicians and healthcare professionals (Lepine and Briley, 2004[55]).

An array of disease conditions where the generation of the neuropathic pain is reported is multiple sclerosis, Guillain-Barre A syndromes (Pentland and Donald, 1994[68]), inflammatory demyelinating polyneuropathies (Albers and Kelly, 1989[6]), non-vasculitic steroid-responsive mononeuritis (Logigian et al., 1993[59]). Damage to the nerve blood vessels and deficiency of pyridoxine (Vitamin B6) could cause peripheral neuropathy (Dellon et al., 2001[20]). To unravel the mechanisms that are responsible for generation and maintenance of neuropathic pain, the development of animal models of neuropathic pain is of great importance (Koltzenburg, 1998[53]). Various animal models have been reported to simulate clinicopathological conditions of peripheral neuropathic, most of which are based on procedures at or near sciatic nerves (Patil et al., 2011[67]).

In animals, partial sciatic nerve ligation (PSNL), i.e. mechanical injury to the sciatic nerve, produces significant damage to myelinated fibers and disruption with loss of unmyelinated axons which results in the appearance of abnormal sensory sign such as allodynia (pain response to low threshold stimulus) and hyperalgesia (increased response to noxious stimuli) which resembles the symptoms that were produced in humans (Bennett and Xie, 1988[10]; Wieczorkiewicz-Plaza et al., 2004[85]). The pain produced after nerve ligation depends upon the degree of nerve trapped in the ligature (Wieczorkiewicz-Plaza et al., 2004[85]). After induction of peripheral nerve injury, along with the appearance of allodynia and hyperalgesia, it also produces an alteration in peptide expression of DRG neurons and dorsal horn (Takaishi et al., 1996[76]).

It has been reported that neutrophils, macrophages, and lymphocytes released from injured cells caused a central neuroimmune response which in turn stimulated activation of microglia and astrocytes (Inoue et al., 1999[32]; Kandhare et al., 2011[38]). This activated glial cell plays a vital role in the subsequent production of cytokines and chemokines (Goswami et al., 2016[27]; Kandhare et al., 2015[40]). The elevated level of proinflammatory cytokines causes release of cyclooxygenase (COX)-2 and substance P which involves in nociceptive transmission (Aswar et al., 2015[7]; Brodin et al., 1986[14]). The elevated levels of substance P caused induction of neuropathic pain following peripheral nerve injury (Kajander and Xu, 1995[35]).

Synthetic chemical moieties like tricyclic anti-depressants (i.e., amitriptyline, nortriptyline, and imipramine), anti-convulsants (i.e., phenytoin, carbamazepine, gabapentin, lamotrigine, and topiramate), pyridoxine (Vitamin B6), thiamine and cyanocobalamin are the present treatment regimens for neuropathic pain (Botez and Herrmann, 2010[11]; Dworkin et al., 2010[22]). However, wide spectrums of adverse effects are associated with these classes of drugs which limit their utility in the management of neuropathic pain (Bril et al., 2011[13]). Also, none of the medications has been found effective in Complex regional pain syndrome (CRPS) during the randomized clinical controlled trials (Kumar et al., 2007[54]; Thomson and Jacques, 2009[77]).

Drugs from herbal origin like Aconiti tuber, Lindera angustifolia, Teucrium polium, Phyllanthus emblica, Vochysia divergens, Cannabis sativa, Nigella sativa, Ocimum sanctum and Ginkgo biloba have been reported preclinically effective for the management of neuropathic pain (Comelli et al., 2008[17]; Kanter, 2008[50]; Kim and Chung, 1992[52]; Muthuraman et al., 2008[63]). Hence, new medicines from herbal origin provide a ray of hope for the treatment of neuropathic pain. 

In Ayurveda, *Azadirachta indica* (Family: Meliaceae) has been reported to have antifungal, antidiabetic, antibacterial, antiviral, contraceptive and sedative properties. It has been evaluated for a wide spectrum of diseases including cancer, inflammation, ulcer, immune disorder, hyperlipidemia and liver disease (Botez and Herrmann, 2010[11]; Chattopadhyay and Bandyopadhyay, 2005[15]; Chattopadhyay, 1996[16]; Dasgupta et al., 2004[18]; Elumalai et al., 2012[24]; Raji et al., 2004[70]). It also possesses to have potential antinociceptive potential (Kanagasanthosh et al., 2016[37]) however, its potential for neuropathic pain has not been evaluated yet. Hence, the aim of the present investigation was to study the effect of *Azadirachta indica* in PSNL induced neuropathic pain in laboratory animals by assessing various nerve functions, biochemical parameters, and molecular parameters.

## Materials and Methods

### Animals

Adult male Wistar rats (180-200 g) were obtained from the National Institute of Biosciences, Pune (India). The animals were housed in groups of 7 in solid bottom polypropylene cages. They were maintained at 24 °C ± 1 °C, with a relative humidity of 45-55 % and 12:12 h dark/light cycle. The acclimation period for animals were two weeks and they were maintained under pathogen-free conditions. The animals had free access to standard pellet chow (Chakan Oil Mills, Sangli) and water throughout the experimental protocol. All experiments were carried out between 09:00 and 17:00 h. The experimental protocols were approved by the Institutional Animal Ethics Committee (IAEC) of Poona College of Pharmacy, Pune (CPCSEA/26/2012) and performed in accordance with the guidelines of Committee for Control and Supervision of Experimentation on Animals (CPCSEA), Government of India on animal experimentation.

### Drugs and chemicals

*Azadirachta indica *leaf standardized extract (4.3 % Total bitters) was purchased from Natural Remedies Pvt. Ltd., Bangalore (Batch No. AI/08001). 1,1',3,3'-Tetraethoxypropane, crystalline beef liver catalase, reduced glutathione (GSH), 5,5'-dithiobis (2-nitrobenzoic acid) were purchased from S.D. Fine Chemicals, Mumbai, India. Total RNA Extraction kit and One-step RT-PCR kit was purchased from MP Biomedicals India Private Limited, India. TNF-α, IL-1β, and NF-κB ELISA kits were purchased from RayBiotech, Inc., Norcross GA. 

### Extraction procedure for Azadirachta indica

Coarse leaves of *Azadirachta indica* were extracted with water and filtered. The filtrate obtained was evaporated and dried under vacuum to obtain a brown powdered extract. The extract thus obtained was analyzed for physicochemical (as per the quality parameters specified in USP 36, [which include analysis of heavy metals] pesticide residues and microbial count) and total bitters. The extract was found to contain 4.3 % bitters by gravimetry. 

### Induction of partial sciatic nerve ligation (PSNL) induced neuropathy and drug treatment schedule

All the required parameters were carried out in all selected rats before surgery. The neuropathic pain was induced in rats by PSNL according to the previously reported procedure (Seltzer et al., 1990[73]). The rats were anesthetized by intraperitoneal injection of Ketamine (80 mg/kg). Rectal temperature was maintained at 37 °C using a homeothermic blanket. The right sciatic nerve was exposed at high thigh level under aseptic condition. The dorsum of the nerve was carefully freed from surrounding connective tissues at a site near the trochanter just distal to the point at which the posterior biceps semitendinosus nerve branches off the common sciatic nerve. A 4-0 silk suture was inserted into the nerve with 3/8 curve, reverse-cutting mini needle and tightly ligated so that the dorsal 1/3 - 1/2 of the nerve thickness was trapped in the ligature. In sham-operated rats, the nerve was left intact (PSNL was not performed). After the surgical procedure, the wounds were closed with 2 muscle sutures and 3-4 skin sutures. In all rats, the left leg and sciatic nerve were remained untouched. Rats were treated with analgesic and antibiotic. Each animal was allowed recovered after surgery for 2 days. 

The animals were divided randomly into groups with 10 rats per group as follows:

**Group I:** Normal group: (N): The rats did not receive either surgery or injury or sciatic nerve. They received only vehicle (Distilled water (DW)).

**Group II:** Sham group: (S): The rats were exposed to the sciatic nerve but did not receive injury. They received only vehicle (DW).

**Group III:** PSNL control group: (PSNL): The rats were exposed to the sciatic nerve and receive injury by PSNL. They received only vehicle (DW).

**Group IV:** Pregabalin treated group: (P(10)): The rats were exposed to the sciatic nerve and received injury. They were treated with Pregabalin at a dose of 10 mg/kg, p.o.

**Group V:** AI (100) treated group: AI (100): The rats were exposed to the sciatic nerve and received injury. They were treated with AI at a low dose of 100 mg/kg, p.o.

**Group VI:** AI (200) treated group: AI (200): The rats were exposed to the sciatic nerve and received injury. They were treated with AI at a medium dose of 200 mg/kg, p.o.

**Group VII:** AI (400) treated group: AI (400): The rats were exposed to the sciatic nerve and received injury. They were treated with AI at a high dose of 400 mg/kg, p.o.

The baseline readings were taken before initiation of the experimentation and drug treatment. The different doses of AI, i.e. 100 mg/kg, 200 mg/kg and 400 mg/kg as well as Pregabalin 10 mg/kg, were administered per oral from day 1 onwards. The treatment continued for 28 days. All the behavioral assays were performed by an observer blind to the drug administration on day 4, 7, 14, 21 and 28 in the morning and doses were administrated immediately afterward. 

At the end of study, i.e. on 28^th ^day, blood was collected from the animals by ROP (retro-orbital puncture) and centrifuged at 7500 rpm for 15 minutes at 4 °C. Then serum was transferred by using micropipette in Eppendorf tubes and stored at 4 °C till analyzed. Then, the animal was sacrificed and sciatic nerve was collected and kept in liquid nitrogen (-70 °C) till analyzed. Few animals sciatic nerve was selected for histopathology evaluation.

### Behavioral tests

Mechanical hyperalgesia (Randall-Selitto paw pressure test), Radiant heat test (Hargreaves test), Thermal hyperalgesia, Mechano-tactile allodynia (Von Frey hair test), Motor incoordination test (Rota-Rod test) and Motor nerve conduction velocity (MNCV) were performed in rats according to previously reported procedures (Kamble et al., 2013[36]; Kandhare et al., 2012[45][46]).

### Measurement of peripheral blood oxygen content

The percentage of hemoglobin saturated with oxygen (pulse Ox) serves as an indicator of *in vivo* peripheral blood oxygen content. Rats were anesthetized with ether and a peripheral pulse Ox sensor (ChoiceMMed, V1.0CF3, MD300CF3, China) was attached to the paw. Pulse Ox readings were taken as the animal regained consciousness (Kandhare et al., 2015[41]). 

### Biochemical estimations

#### Sciatic nerve homogenate preparation

50 mg sciatic nerve samples were rinsed with ice cold saline (0.9 % sodium chloride), minced and homogenized at 3000 rpm in chilled tris buffer (10 mM, pH 7.4) and diluted up to 5 ml. The homogenates were centrifuged at 10,000 g at 0 °C for 20 min. It was divided into aliquot to determine superoxide dismutase (SOD), glutathione (GSH), malondialdehyde (MDA), total calcium content, nitric oxide content, membrane-bound inorganic phosphate enzyme, tumor necrosis factor-alpha (TNF-α), interleukin (IL)-1β and nuclear factor-kappaB (NF-κB) according to previously reported procedures (Adil et al., 2016[1][2]; Kandhare et al., 2016[39], 2012[48]; Visnagri et al., 2015[79]).

#### Estimation of TNF-α, IL-1β, and NF-κB

The quantifications of TNF-α, IL-1β, and NF-κB were performed with the help and instructions provided by Thermo Scientific, USA Rat TNF-α, IL-1β and NF-κB immunoassay kit (Kandhare et al., 2013[43]; Sarkate et al., 2015[72]; Visnagri et al., 2015[78]). The sample values are then read off the standard curve. Values were expressed as means ± S.E.M. 

#### Reverse transcriptase PCR 

The levels of mRNA were analyzed in sciatic nerve using a reverse transcription (RT)- Polymerase chain reaction (PCR) approach as described previously (Adil et al., 2016[3], 2014[5]). Single-stranded cDNA was synthesized from 5 µg of total cellular RNA using reverse transcriptase (MP Biomedicals India Private Limited, India) as described previously (Adil et al., 2014[5]; Ketkar et al., 2015[51]). The primer sequence for Bax, Bcl-2, Caspase-3, iNOs and β-actin is presented in Supplementary Table 1. Expression of all the genes was assessed by generating densitometry data for band intensities in different sets of experiments and was generated by analyzing the gel images on the Image J program (Version 1.33, Wayne Rasband, NIH, Bethesda, MD, USA) semi-quantitatively. The band intensities were compared with constitutively expressed β-actin. The intensity of mRNAs were standardized against that of the β-actin mRNA from each sample, and the results were expressed as PCR-product/β-actin mRNA ratio.

#### Preparation of single Schwann cell (SC) suspensions

Preparation of single Schwann cell (SC) suspensions and determination of apoptotic cell populations were determined as previously described (Kandhare et al., 2012[45]). At the end of treatment, the sciatic nerves of rats were collected and mixed with 0.4 % collagenase and 0.25 % Trypsin at 370 °C for 30 min and dissociated, ground and the obtained homogenate was passed through a 70 μm nylon mesh. Single SC suspension was washed three times with phosphate-buffered saline (PBS).

#### Flow cytometry analysis

In order to determine SC apoptosis, the isolated SC were incubated with rabbit anti-cow S-100 antibody and followed by staining with APC-goat anti-rabbit IgG (both from BD) with Fluorescein isothiocyanate (FITC)-Annexin V and PI (Sigma). The percentages of expression of Fas and Annexin-Von gated S-100 positive SC were analyzed by an FACSCalibur cytometer using CellQuest software (Becton & Dickinson, San Diego, USA) (Kandhare et al., 2014[40]).

#### ROS production in SC by H_2_DCFDA probe using Flow Cytometry

Reactive Oxygen Species (ROS) production was quantified by using dichlorodihydrofluorescein diacetate (H_2_DCFDA) dye method based on the ROS-dependent oxidation of DCFH-DA to DCF according to the method described elsewhere (Ghule et al., 2015[26]). 

### Histopathological analysis

On day 28^th^ all animals were sacrificed and sciatic nerves were collected. Samples of sciatic nerve was kept in the fixative solution (10 % formalin) and it was processed for 12 hr using isopropyl alcohol, xylene, and paraffin embedded for light microscopic study (Nikon E200). Paraffin-embedded tissue section cut at 5 μm thickness were prepared and staining was done by using hematoxylin and eosin. Nerve sections were analyzed qualitatively under a light microscope (400 X) for axonal degeneration, hemosiderin deposition, edema, neutrophil infiltration, nerve cell vacuolization, etc.

### Statistical analysis

Data analysis was performed using GraphPad Prism 5.0 software (GraphPad, San Diego, USA). Statistical comparisons were made between drug-treated groups and disease control animals (PSNL control). Data of disease activity index were statistically analyzed using two-way repeated ANOVA, Bonferroni's multiple range test was applied for post hoc analysis, while data of biochemical parameters were analyzed using one-way ANOVA, Tukey's multiple range test was applied for post hoc analysis. A value of *p < *0.05 was considered to be statistically significant.

## Result

### Effect of AI on PSNL induced alterations in mechanical allodynia in von Frey hair test and mechanical hyperalgesia in Randall-Selitto paw pressure test

**Figure 2:**
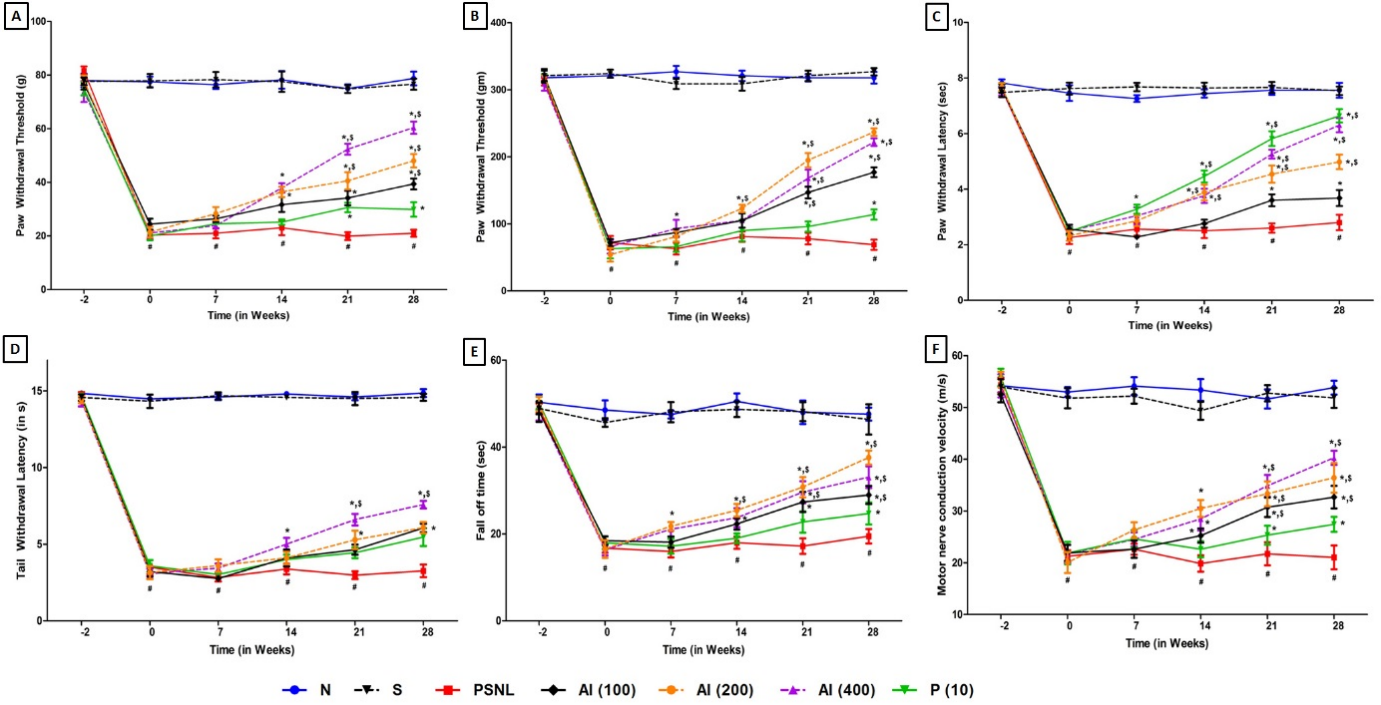
Effect of Azadirachta indica on PSNL-induced alterations in (A) Mechanical allodynia in von Frey hair test, (B) Mechanical hyperalgesia in paw pressure test, (C) Thermal hyperalgesia in plantar test, (D) Thermal hyperalgesia in Hargreaves test, (E) fall off time in rotarod test and (F) Motor nerve conduction velocity. Data are expressed as mean ± S.E.M. from six rats and analyze by two-way ANOVA followed by Bonferroni's test. *p < 0.05 as compared to PSNL control group, #p < 0.05 as compared to normal and sham control animals and $p < 0.05 as compared to one another group. N: Normal rats; S: Sham control rats; PSNL: Partial Sciatic Nerve Ligation control rats, P (10): Pregabalin (10 mg/kg, p.o.) treated rats, AI (100): Azadirachta indica (100 mg/kg, p.o.) treated rats, AI (200): Azadirachta indica (200 mg/kg, p.o.) treated rats and AI (400): Azadirachta indica (400 mg/kg, p.o.) treated rats.

The mean paw-withdrawal threshold of normal rats as well as sham control rats before the induction of neuropathy did not significantly differ as compared to PSNL control rats. Partial sciatic nerve ligation resulted in significant decreased (*p < *0.05) mechano-tactile allodynia and mechanical hyperalgesia, i.e. mean paw withdrawal threshold in PSNL control rats as compared to sham-operated rats. Chronic treatment with AI (100, 200 and 400 mg/kg, p.o.) for 28 days significantly increased (*p < *0.05) paw withdrawal threshold when compared with PSNL control rats. Administration of pregabalin (10 mg/kg, p.o.) also significantly increased (*p < *0.05) mechanical nociceptive threshold as compared to PSNL control rats. When compared with pregabalin (10 mg/kg, p.o.) treated rats, AI (100, 200 and 400 mg/kg, p.o.) treated rats showed a more significant increase (*p < *0.05) in mechanical allodynia and hyperalgesia. However, there was no significant change in mean paw withdrawal threshold in normal as well as sham control group over the same time period (Figure 2A, B(Fig. 2)).

### Effect of AI on PSNL induced alterations in thermal hyperalgesia in paw withdrawal latency and tail withdrawal latency in Hargreaves test and tail immersion test

Before the induction of neuropathy, there was no significant difference in mean paw as well as tail withdrawal latency i.e. reaction time in normal rats as compared to PSNL control rats as well as sham control rats. A significant decrease (*p < *0.05) in paw withdrawal latency and tail withdrawal latency as produced in the PSNL control rats after induction of neuropathy on day 0 was seen as compared to sham-operated rats. Chronic administration of the AI (100, 200 and 400 mg/kg, p.o.) for 28 days significantly increased (*p < *0.05) the paw withdrawal latency and tail withdrawal latency as compared to PSNL control rats. Administration of pregabalin (10 mg/kg, p.o.) also significantly increased (*p < *0.05) paw and tail withdrawal latency as compared to PSNL control rats. There was no significant change in paw withdrawal latency as well as a tail withdrawal latency in normal as well as sham control group over the same time period (Figure 2C, D(Fig. 2)).

### Effect of AI on PSNL induced alterations in motor incoordination test

There was no significant difference in fall off time in PSNL control rats as compared to normal rats as well as sham control rats before the induction of neuropathy. Induction of neuropathy by partial sciatic nerve ligation resulted in significant decrease in fall off time, i.e. motor incoordination in PSNL control rats as compared to sham-operated rats on day 0. Chronic administration of AI (100, 200 and 400 mg/kg, p.o.) for 28 days significantly attenuated (*p < *0.05) these partial sciatic nerve ligation-induced a decrease in motor performance i.e. mean fall off time as compared to PSNL control rats. When compared with PSNL control rats, pregabalin (10 mg/kg, p.o.) treatment showed a significant increase (*p < *0.05) in motor performance. Sham control rats did not show any significant alterations in fall off time as compared to normal rats over the same time period (Figure 2E(Fig. 2)).

### Effect of AI on PSNL induced alterations in motor nerve conduction velocity

Motor nerve conduction velocity did not differ significantly in PSNL control rats as compared to sham control or normal rats before induction of neuropathy on day 0. Partial sciatic nerve ligation resulted in significant decrease (*p < *0.05) in motor nerve conduction velocity in PSNL control rats as compared to sham-operated rats. Chronic administration of AI (100, 200 and 400 mg/kg, p.o.) for 28 days resulted in a significant increase (*p < *0.05) in motor nerve conduction velocity as compared to PSNL control rats. Treatment with pregabalin (10 mg/kg, p.o.) also showed a significant increase (*p < *0.05) in motor nerve conduction velocity as compared to PSNL control rats. Motor nerve conduction velocity did not differ significantly in normal rats as compared to sham control rats over the same time period (Figure 2F(Fig. 2)).

### Effect of AI on PSNL induced alterations in neural oxido-nitrosative level

Induction of neuropathy by PSNL resulted in a significant decrease (*p < *0.05) in SOD and GSH level in sciatic nerve of PSNL control rats as compared to sham control rats and normal rats. After chronic administration of AI (200 and 400 mg/kg, p.o.) for 28 days, the SOD and GSH level was significantly increased when compared with PSNL control rats (Table 1(Tab. 1)). 

Partial sciatic nerve ligation resulted in significant increase (*p < *0.05) in MDA and NO level in sciatic nerve of PSNL control rats as compared to sham operated and normal rats. After 28 days of AI treatment (200 and 400 mg/kg, p.o.), MDA and NO level were decreased significantly as compared to PSNL control rats. Administration of pregabalin (10 mg/kg, p.o.) also significantly inhibited PSNL induced alteration in neural oxido-nitrosative level when compared with PSNL control rats. However, there was no significant difference in the SOD, GSH, MDA and NO level of sham control rats as compared to normal rats (Table 1(Tab. 1)).

### Effect of AI on PSNL induced alterations in neural calcium and Na-K-ATPase level 

Partial sciatic nerve ligation resulted in a significant increase (*p < *0.05) in neural calcium level whereas it significantly decreased in Na-K-ATPase level in PSNL control rats when compared with sham-operated rats and normal rats. The 28-day treatment of AI (200 and 400 mg/kg, p.o.) significantly decreased (*p < *0.05) in neural calcium level as compared to PSNL control rats. Na-K-ATPase level was increased significantly (*p < *0.05) in AI (200 and 400 mg/kg, p.o.) treated rats when compared with PSNL control rats. Pregabalin (10 mg/kg, p.o.) treatment significantly increased (*p < *0.05) neural Na-K-ATPase level and significantly decreased (*p < *0.05) neural calcium level as compared to PSNL control rats (Table 1(Tab. 1)).

### Effect of AI on PSNL induced alterations in peripheral blood oxygen content

After 28 day duration of induction of neuropathy by partial sciatic nerve ligation, peripheral blood oxygen content of PSNL control rats significantly decreased (*p < *0.05) as compared to normal rats as well as sham-operated rats. After 28 days of chronic treatment with AI (200 and 400 mg/kg, p.o.), peripheral blood oxygen content was significantly increased (*p < *0.05) as compared to PSNL control rats. PSNL induced decrease in peripheral blood oxygen was significantly increased (*p < *0.05) by pregabalin (10 mg/kg, p.o.) treatment. Peripheral blood oxygen content did not differ significantly in normal rats as well as sham control rats over the same time period (Table 1(Tab. 1)).

### Effect of AI on PSNL induced alterations in neural TNF-α, IL-1β, and NF-κB levels

TNF-α, IL-1β and NF-κB levels in sciatic nerve of PSNL control rats were significantly increased (*p < *0.05) after induction of neuropathy as compared to normal as well as sham control rats. Chronic treatment with AI (200 and 400 mg/kg, p.o.) resulted in significant decrease (*p < *0.05) in elevated neural TNF-α, IL-1β and NF-κB level as compared to PSNL control rats. Pregabalin (10 mg/kg, p.o.) treatment significantly decreased elevated neural TNF-α, IL-1β and NF-κB levels as compared to PSNL control rats. There was no significant difference in neural TNF-α, IL-1β and NF-κB levels of sham control rats as compared to normal rats over the same time period (Table 2(Tab. 2)).

### Effect of AI on PSNL induced alterations in neural Bax, Bcl-2, Caspase-3 and iNOs mRNA level 

**Figure 3:**
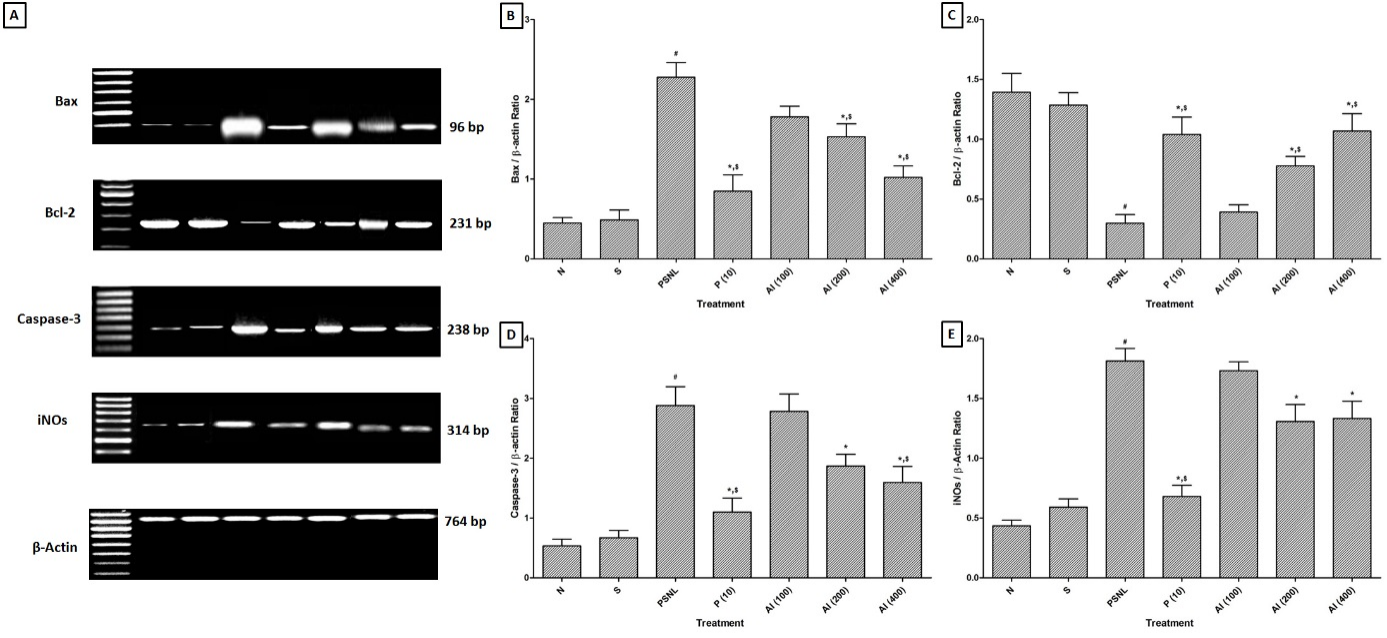
Effect of *Azadirachta indica* on PSNL-induced alterations in mRNA expression of Bax, Bcl-2, Caspase-3 and iNOs in sciatic nerve as determined by relative quantification by reverse transcriptase polymerase chain reaction analysis (A), Quantitative representation of mRNA expression of Bax, Bcl-2, Caspase-3 and iNOs (B-E). Data are expressed as mean ± S.E.M. from six rats and were analyzed by One-way ANOVA followed by Tukey's test. *p < 0.05 as compared to PSNL control group, ^#^*p < *0.05 as compared to normal and sham control animals and ^$^*p < *0.05 as compared to one another group. N: Normal rats; S: Sham control rats; PSNL: Partial Sciatic Nerve Ligation control rats, P (10): Pregabalin (10 mg/kg, p.o.) treated rats, AI (100): *Azadirachta indica* (100 mg/kg, p.o.) treated rats, AI (200): *Azadirachta indica* (200 mg/kg, p.o.) treated rats and AI (400): *Azadirachta indica* (400 mg/kg, p.o.) treated rats.

Partial sciatic nerve ligation resulted in significant up-regulation (*p < *0.05) in neural Bax, Caspase-3 and iNOs mRNA expression level as significant down-regulation in neural Bcl-2 mRNA expression in PSNL control rats as compared to sham-operated as well as normal rats. After 28 days of AI (200 and 400 mg/kg, p.o.) treatment, neural Bax, Caspase-3, and iNOs mRNA expression level were significantly down-regulated (*p < *0.05) as compared to PSNL control rats. The down-regulated neural Bcl-2 mRNA expression was significantly up-regulated by AI (200 and 400 mg/kg, p.o.) treatment as compared to PSNL control rats. The altered neural Bax, Bcl-2, Caspase-3 and iNOs mRNA levels were significantly attenuated by pregabalin (10 mg/kg, p.o.) treatment as compared to PSNL control rats (Figure 3(Fig. 3)).

### Effect of AI on PSNL induced alterations in neural apoptosis and ROS levels

**Figure 4:**
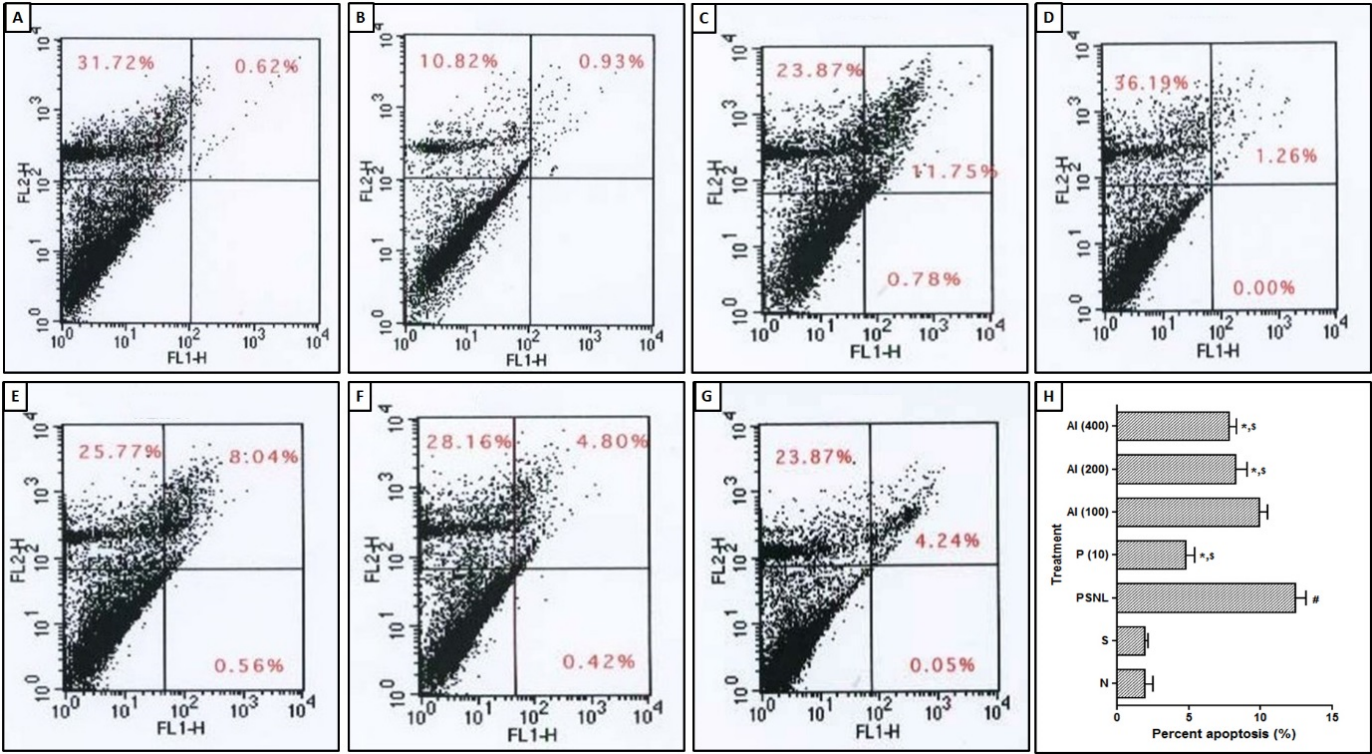
Effect of *Azadirachta indica* on PSNL-induced alterations in neural apoptosis. Representative image of FACS analysis using Annexin V/FITCPI stain (A-G) and percentage necrotic cells population (G). Four distinct cell distribution patterns are visible: normal viable cells (lower left quadrant), apoptotic cells (lower right quadrant), late apoptotic or necrotic cells (upper right quadrant) and necrotic cells (upper left quadrant). Data are expressed as mean ± S.E.M. from six rats and were analyzed by One-way ANOVA followed by Tukey's test. *p < 0.05 as compared to PSNL control group, ^#^*p < *0.05 as compared to normal and sham control animals and ^$^*p < *0.05 as compared to one another group. N: Normal rats; S: Sham control rats; PSNL: Partial Sciatic Nerve Ligation control rats, P (10): Pregabalin (10 mg/kg, p.o.) treated rats, AI (100): *Azadirachta indica* (100 mg/kg, p.o.) treated rats, AI (200): *Azadirachta indica* (200 mg/kg, p.o.) treated rats and AI (400): *Azadirachta indica* (400 mg/kg, p.o.) treated rats.

**Figure 5:**
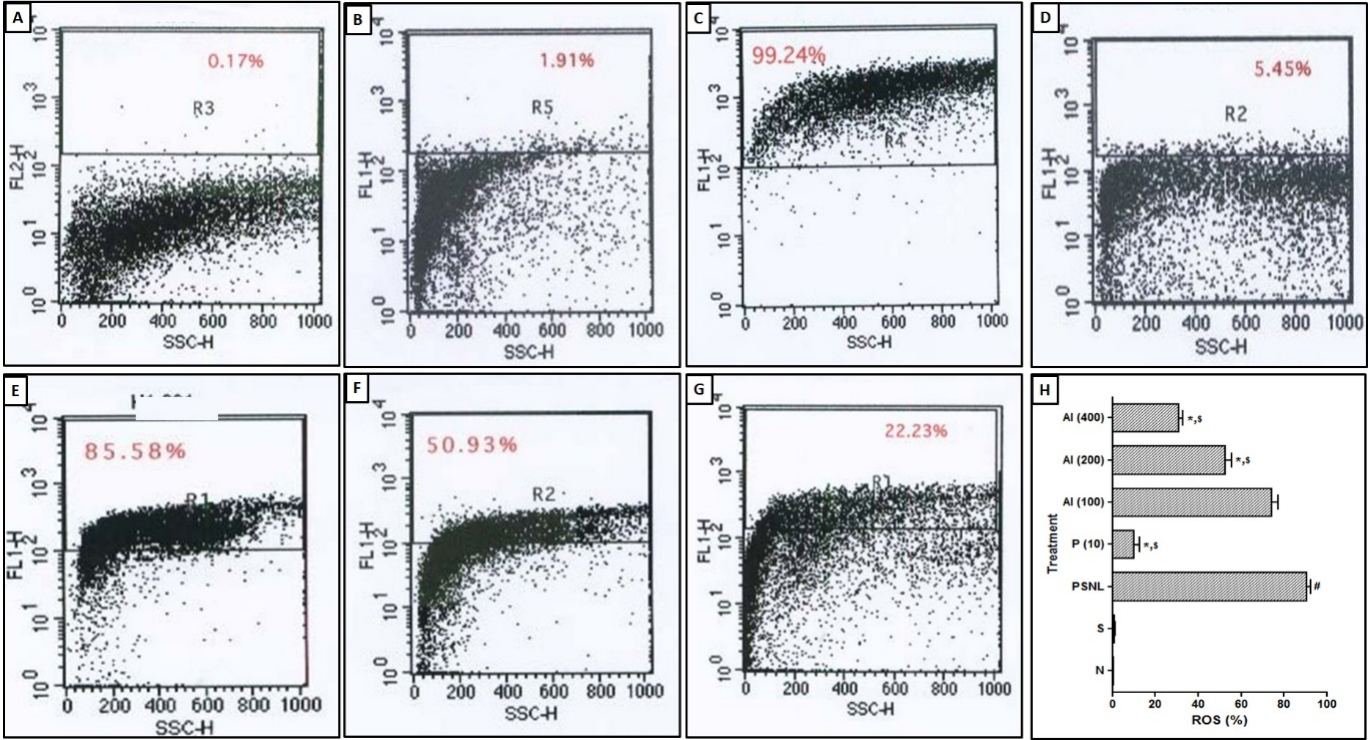
Effect of *Azadirachta indica* on PSNL-induced alterations in neural ROS generation. Representative images of FACS analysis using H_2_DCFD stain (A-E) and percentage ROS (G). Data are expressed as mean ± S.E.M. from six rats and were analyzed by One-way ANOVA followed by Tukey's test. *p < 0.05 as compared to PSNL control group, ^#^*p < *0.05 as compared to normal and sham control animals and ^$^*p < *0.05 as compared to one another group. N: Normal rats; S: Sham control rats; PSNL: Partial Sciatic Nerve Ligation control rats, P (10): Pregabalin (10 mg/kg, p.o.) treated rats, AI (100): *Azadirachta indica* (100 mg/kg, p.o.) treated rats, AI (200): *Azadirachta indica* (200 mg/kg, p.o.) treated rats and AI (400): *Azadirachta indica* (400 mg/kg, p.o.) treated rats.

Apoptosis and ROS in sciatic nerve of PSNL control rats were significantly increased as compared to sham-operated as well as normal rats. PSNL induced increase in apoptosis and ROS was significantly decreased by AI (200 and 400 mg/kg) treatment as compared to PSNL control rats. Rats treated with pregabalin (10 mg/kg, p.o.) also significantly inhibited this elevated level of apoptosis and ROS as compared to PSNL control rats (Figure 4(Fig. 4) and Figure 5(Fig. 5)).

### Effect of AI on the histopathological alteration of sciatic nerve caused by PSNL

**Figure 6:**
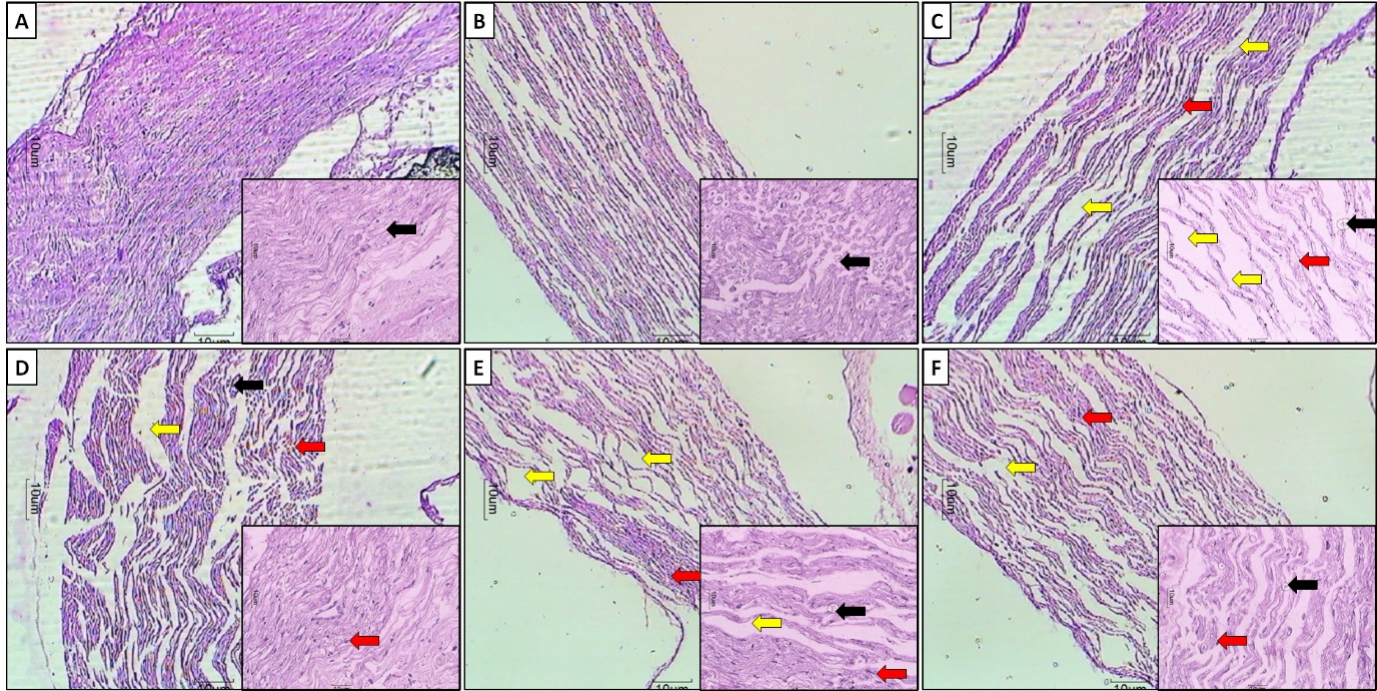
Effect of *Azadirachta indica* on PSNL-induced alterations in the histopathology of the sciatic nerve. Photomicrographs of sections of sciatic nerve from rats stained with H & E. Sciatic nerve microscopic image of (A) Normal rats; (B) Sham control rats; (C) Partial Sciatic Nerve Ligation control rats, (D) Pregabalin (10 mg/kg, p.o.) treated rats, (E) *Azadirachta indica* (200 mg/kg, p.o.) treated rats and (F) *Azadirachta indica* (400 mg/kg, p.o.) treated rats.

Figure 6A and B(Fig. 6) depicted the normal architecture of sciatic nerve from normal and sham control rats. It's devoid of any infiltration of neutrophils, edema, nerve cell vacuolization and necrosis. Partial nerve ligation caused histopathological alterations in sciatic nerve of PSNL control rats. Histological evaluation of sciatic nerve from PSNL control rats showed axonal degeneration evident by the decrease in a number of myelinated fibers along with swelling of nonmyelinated and myelinated nerve fibers. It also showed infiltration of neutrophils and lymphocytes (red arrow) in the nerve tissues (Figure 6C(Fig. 6)). Also, fiber dearrangement was noted and showed an elevated level of intracellular edema formation (black arrow) along with tissue necrosis (yellow arrow). However, administration of AI (200 and 400 mg/kg) attenuated PSNL induced axonal degeneration and histopathological alterations. It shows mild lymphocytic infiltration (red arrow) and edema (black arrow) (Figure 6E and F(Fig. 6)). Figure 6D(Fig. 6) depicted the histopathological architecture of sciatic nerve tissues from pregabalin (10 mg/kg, p.o.) treated rats reflected attenuation of PSNL-induced neutrophilic as well as lymphocytic infiltration, edema, and necrosis in the nerve fibers (Table 3(Tab. 3)).

## Discussion

Partial sciatic nerve ligation model of peripheral neuropathic pain mimics most of the important characteristic features of neurogenic pain in patients after peripheral nerve injury, therefore, it is widely accepted and a widely used model of peripheral neuropathic pain (Jain et al., 2009[33]; Muthuraman et al., 2008[63]). 

It has been documented that sensitization of primary afferent nerves is responsible for generation and maintenance of hyperalgesia (Kim and Chung, 1992[52]). During chronic neuropathic pain, there is an elevated level of potassium ion and substance P in spinal C-fiber that caused decrease in stimulation threshold of physical and thermal receptors (Levy and Zochodne, 2000[56]). Unilateral partial sciatic nerve ligation resulted in a decrease of mechanical allodynia and thermal hyperalgesia ipsilaterally which produced within post hours of surgery and are maintained for months. Our results are in coincidence with the previous finding (Kim and Chung, 1992[52]) where unilateral partial sciatic nerve ligation resulted in inflammation which may be due to the release of inflammatory mediators such as bradykinin, nitric oxide, and a gene-related peptide calcitonin. However, chronic treatment with AI significantly attenuated decreased level of mechanical allodynia and thermal hyperalgesia in sciatic nerve ligated animals indicating its therapeutic potential against neuropathic pain. The results of the present investigation coincided with the earlier works which proved credence to the analgesic and anti-inflammatory activity of *Azadirachta indica* (Srinivasa et al., 2014[75]). The findings of the present investigation suggest that AI plays an important role in pain regulation at the peripheral level. 

Central sensitization of neurons is a result of damages of the motor as well as sensory fibers due to partial sciatic nerve ligation thus causes a decrease in thermal withdrawal latency. Tail flick method is a useful technique for the evaluation of peripherally as well as centrally acting analgesic drugs (Wang et al., 2005[83]). In the present study, we were able to confirm this thermal hyperalgesia using an independent measure of the force of hind limb withdrawal elicited by graded thermal stimuli. Treatment with AI significantly increased tail withdrawal latency in partial sciatic nerve ligated rats.

There is increasing evidence that PSNL was associated with elevated level of neural calcium (Jain et al., 2009[33]). The rise in calcium level stimulates generation of secondary messengers, i.e. activation of calcium binding protein as well as calcium dependent kinase and phosphatase action. It caused a disturbance in the normal homeostasis function of the nervous system as well as increased auto-destruction with long-term potentiation, long-term depression and neuronal hyperexcitation (Young, 1992[87]). It has been documented that activated calpains and calmodulin was responsible for causing alteration of stability of axonal cytoskeleton protein thereby axonal degeneration (Raygude et al., 2012[71]; Visnagri et al., 2014[80]). Chronic treatment with AI exerts its neuroprotective effect via attenuating the elevated level of neuronal calcium.

PSNL is associated with the generation of a vicious cycle that comprises of release of toxic substances from neutrophils and macrophages leading to generation and accumulation of reactive oxygen species (ROS) as well as inflammatory cytokines (Bridges et al., 2001[12]). Furthermore, nitric oxide (NO) is an unconventional intracellular messenger in the nervous system and its elevated level associated with sensitized spinal as well as central neurons (Lin et al., 1999[58]). NO reacts with ROS and acts as an oxidant (Adil et al., 2015[4]; Badole et al., 2015[9]; Kamble et al., 2013[36]). Reaction with deficient superoxide dismutase leads to the production of notorious peroxynitrite, which affects any cell molecule (Honmore et al., 2015[29], 2016[30]). The increase in NO level is the result of tissue toxicity caused by damage of nerve. In the present investigation, the level of nitric oxide (NO) was increased due to injury to sciatic nerve which concerned in neuropathic pain (Levy and Zochodne, 2004[57]). Treatment of AI inhibited the elevated level of oxido-nitrosative stress in sciatic nerve and elucidates its neuroprotective effect. The finding of the present investigation was in line with previously published experimental evidence (Dasgupta et al., 2004[18]).

Previously it has been shown that a conspicuous inflammatory reaction, an immune cell infiltration and increased endoneurial levels of pro-inflammatory cytokines (including TNF-α, interleukin-1β, and interleukin-6) detected at the site of nerve injury in animal models of painful peripheral neuropathy (DeLeo et al., 1997[19]). TNF-α and IL-1β as important mediators of neuropathic pain play an important role in initiation and maintenance of neuropathic pain (Ignatowski et al., 1999[31]). Nuclear factor kappa B (NF-κB) is a transcription factor that can be activated by these cytokines (Aswar et al., 2015[7]; Kandhare et al., 2012[47]). It plays a crucial role during inflammation, injury and neuronal plasticity for glial and neuronal cell (Ma and Bisby, 1998[60]). In the present investigation injury to sciatic nerve causes an increase in levels of pro-inflammatory cytokines including TNF-α and IL-1β. Our finding provides credence to the previous finding (Gustafson-Vickers et al., 2008[28]). Treatment with AI inhibited the release of inflammatory cytokine and thus NF-κB which may be due to the virtue of its anti-inflammatory property and reversed the neuropathic pain. 

It is well known that membrane-bound enzyme Na^+^K^+^ATPase plays an important role in the generation of the transmembraneous Na^+^K^+^ gradient by catalyzing the hydrolysis of ATP (Patil et al., 2015[66]; Visnagri et al., 2013[81]). The physiochemical state of the lipid environment regulates the activity of these membrane-bound enzymes. The increase in membrane fluidity caused enhanced activity of Na^+^K^+^ATPase (Visnagri et al., 2012[82]). The decrease in Na^+^K^+^ATPase in sciatic nerve homogenate indicated failure of the Na-K-pump, which required to expel intracellular Na ions (Mohod et al., 2016[61]; Mukherjee et al., 2015[62]). The increase in pain pursued by animals is due to failure of Na^+^K^+^ATPase leading to neuropathic symptoms in the animals. The results of the present study indicated AI treatment significantly restores decrease level of Na^+^K^+^ATPase which was localized and distributed on the cytoplasmic side of the axonal membrane, confirming the role of this enzyme in the transmission of electrical signals.

PSNL affects impulse generation along with alterations in the pattern of action potential activity (Kandhare et al., 2012[45]). Furthermore, pathological characteristics of ligated sciatic nerve include various abnormalities including loss of myelinated nerve fiber, axonal degeneration. Axonal degeneration of peripheral nerves leads to dysfunction of Schwann cells that resulted in delayed MNCV in PSNL control animals. The histopathological results also support these findings where sciatic nerve from PSNL control rats showed loss of myelinated nerve fiber. Furthermore, demyelinated nerve injury leads to a significant decrease in the percentage of hemoglobin oxygen saturation (Radhakrishnan et al., 2006[69]). The pulse Ox investigation in the present study showed that treatment with AI caused a significant increase in the percentage of hemoglobin oxygen saturation which in turn enhances nerve conduction velocity via decreasing pathological damage.

Cell apoptosis in integral part of various neurodegenerative conditions and inducible nitric oxide synthase (iNOS) has been reported to play a vital role in either apoptosis or necrosis (Devkar et al., 2016[21]; Kandhare et al., 2015[44]; Pannu and Singh, 2006[65]). Furthermore, the balance between Bcl-2-associated X protein (Bax) and B-cell lymphoma 2 (Bcl-2) has an important factor in cell survival (Kandhare et al., 2015[42]; Oltvai et al., 1993[64]). Literature punctuated with the evidence showed that sciatic nerve of PSNL rats associated with decreased Bcl-2 expression. The researcher reported that prior to apoptotic cell death, caspase signaling is responsible for generation and maintenance of the pain in the small fiber peripheral neuropathy (Joseph and Levine, 2004[34]). Caspase-3 plays a vital role during the apoptosis via cleavage of caspase-related proteins (Adil et al., 2015[4]; Kandhare et al., 2015[44]). In the present study, we also found up-regulated mRNA expressions of Bax and caspase-3 whereas down-regulated Bcl-2 mRNA expression in PSNL control rats. Treatment with AI significantly prevented PSNL induced alterations in these apoptotic mRNA expressions in sciatic nerve. Flow cytometric analysis also supports these findings where the population of early apoptotic cells was decreased after AI treatment. Results of the present investigation corroborate with the findings of previous investigator depicting anti-apoptotic potential of AI (Elumalai et al., 2012[24]).

Recently some herbal drugs have been reported clinically for attenuation of neuropathic pain successfully (Babbar et al., 2009[8]; Ellis et al., 2009[23]). Patient receiving single inhalation of tetrahydrocannabinol (25 mg, three times daily for five days) has been shown to reduce the intensity of pain (Ware et al., 2010[84]). Hence, our finding suggests that *Azadirachta indica* elucidates its neuroprotective effect in partial sciatic nerve ligation-induced neuropathic pain by virtue of its antioxidant, anti-inflammatory, anti-apoptotic potential. 

## Conflict of interest

The authors declare that there are no conflicts of interest.

## Acknowledgements

The authors acknowledge Dr. S. S. Kadam, Vice-chancellor, and Dr. K. R. Mahadik, Principal, Poona College of Pharmacy, Bharati Vidyapeeth Deemed University, Pune, India, for providing the necessary facilities to carry out the study. 

## Grants, sponsors, and funding sources

None.

## Supplementary Material

Supplementary material

## Figures and Tables

**Table 1:**
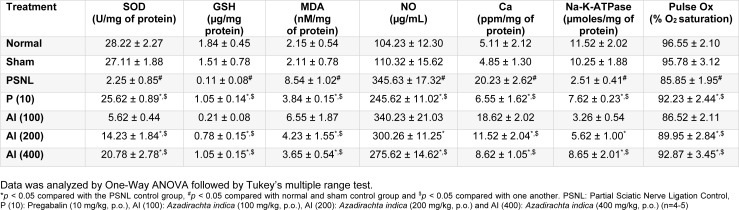
Effect of Azadirachta indica on PSNL-induced alterations in neural SOD, GSH, MDA, NO, Calcium, Na-K-ATPase and Pulse Ox levels

**Table 2:**
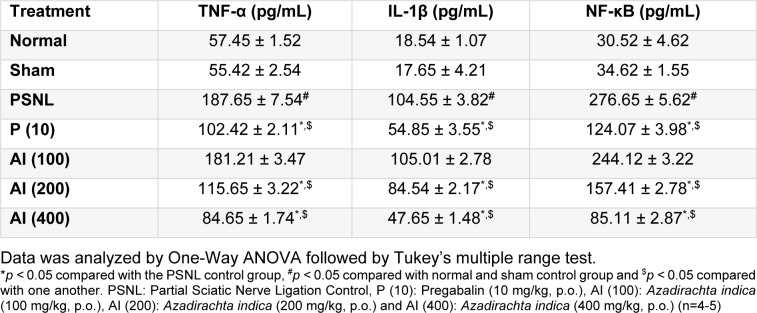
Effect of *Azadirachta indica* on PSNL-induced alterations in neural TNF-α, IL-1β, and NF-κB levels

**Table 3:**
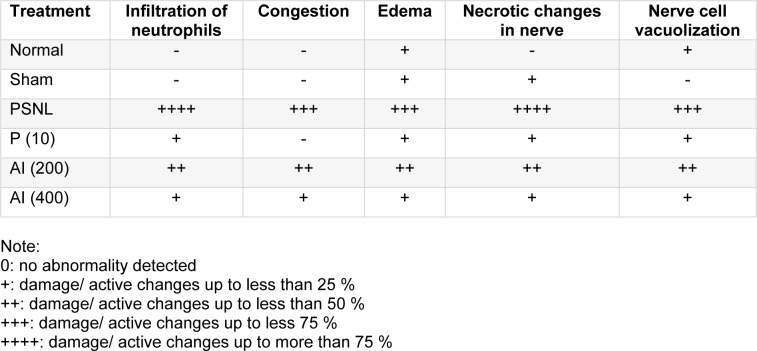
Effect of *Azadirachta indica* on PSNL-induced alterations in histopathology of sciatic nerve in rats

**Figure 1:**
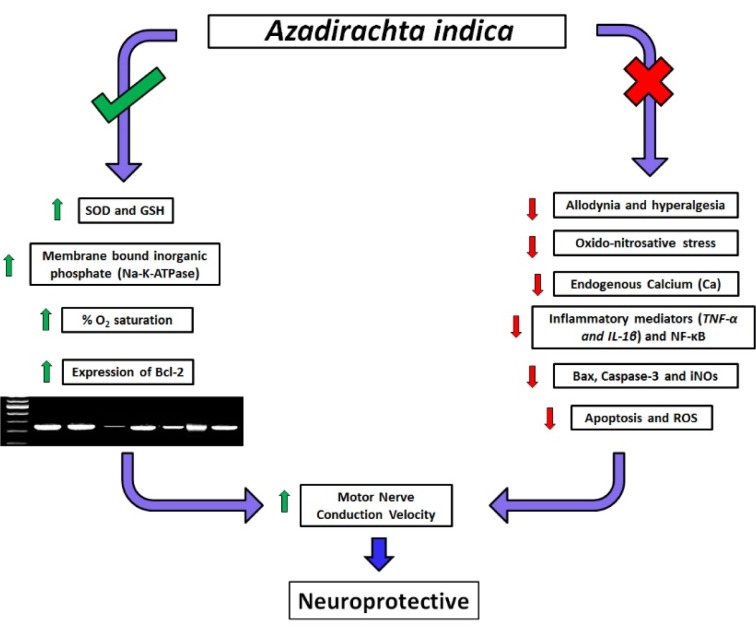
Graphical abstract
