# Supplementary material for: Neuroprotective effect of Azadirachta indica standardized extract in partial sciatic nerve injury in rats: Evidence from anti-inflammatory, antioxidant and anti-apoptotic studies
Source: EXCLI J. 2017 Apr 18;16:546–65. doi: 10.17179/excli2017-161 (PMC5491907; doi:10.17179/excli2017-161)
Supplement: Supplementary material [file EXCLI-16-546-s-001.pdf]

EXCLI Journal 2017;16:546-565 – ISSN 1611-2156
Received: January 21, 2017, accepted: March 22, 2017, published: April 18, 2017

Supplementary information to

NEUROPROTECTIVE EFFECT OF *AZADIRACHTA INDICA* STANDARDIZED EXTRACT IN PARTIAL SCIATIC NERVE INJURY IN RATS: EVIDENCE FROM ANTI-INFLAMMATORY, ANTIOXIDANT AND ANTI-APOPTOTIC STUDIES

Amit D. Kandhare, Anwesha A. Mukherjee, Subhash L. Bodhankar*

Department of Pharmacology, Poona College of Pharmacy,
Bharati Vidyapeeth Deemed University, Erandwane, Paud Road, Pune-411 038, India

* corresponding author: Dr. S. L. Bodhankar, Dept. of Pharmacology, Poona College of Pharmacy, Bharati Vidyapeeth Deemed University, Erandwane, Pune-411038, Maharashtra; E-mail: drslbodh@gmail.com

http://dx.doi.org/10.17179/excli2017-161

This is an Open Access article distributed under the terms of the Creative Commons Attribution License (http://creativecommons.org/licenses/by/4.0/).

Supplementary Table 1: Primer sequences for Bax, Bcl-2, Caspase-3, iNOs and β-actin

**Sr. No.**	**Gene**	**Primer Sequence (5'-3')**		**Size (bp)**
		**Forward primer**	**Reverse primer**	
1	Bax	GGGAATTCTGGAGCTGCAGAGGATGATT	GCGGATCCAAGTTGCCATCAGCAAACAT	96
2	Bcl-2	CTGTACGGCCCCAGCATGGCG	GCTTTGTTTCATGGTACATC	231
3	Caspase-3	CTCGGTCTGGTACAGATGTCGATG	GGTTAACCCGGGTAAGAATGTGCA	238
4	iNOs	ATCCCGAAACGCTACACTT	TCTGGCGAAGAACAAATCC	314
5	β-actin	GTCACCCACACTGTGCCCATCT	ACAGAGTACTTGCGCTCAGGAG	764
